# Can electric fields drive chemistry for an aqueous microdroplet?

**DOI:** 10.1038/s41467-021-27941-x

**Published:** 2022-01-12

**Authors:** Hongxia Hao, Itai Leven, Teresa Head-Gordon

**Affiliations:** 1 Chemical Sciences Division, Lawrence Berkeley National Laboratory, University of California, Berkeley, CA 94720 USA; 2 Pitzer Center for Theoretical Chemistry, University of California, Berkeley, CA 94720 USA; 3 Departments of Chemistry, University of California, Berkeley, CA 94720 USA; 4 Departments of Bioengineering, University of California, Berkeley, CA 94720 USA; 5 Departments of Chemical and Biomolecular Engineering, University of California, Berkeley, CA 94720 USA

**Keywords:** Catalysis, Surface chemistry, Computational chemistry, Molecular dynamics

## Abstract

Reaction rates of common organic reactions have been reported to increase by one to six orders of magnitude in aqueous microdroplets compared to bulk solution, but the reasons for the rate acceleration are poorly understood. Using a coarse-grained electron model that describes structural organization and electron densities for water droplets without the expense of ab initio methods, we investigate the electric field distributions at the air-water interface to understand the origin of surface reactivity. We find that electric field alignments along free O–H bonds at the surface are ~16 MV/cm larger on average than that found for O–H bonds in the interior of the water droplet. Furthermore, electric field distributions can be an order of magnitude larger than the average due to non-linear coupling of intramolecular solvent polarization with intermolecular solvent modes which may contribute to even greater surface reactivity for weakening or breaking chemical bonds at the droplet surface.

## Abstract (web summary)

Theoretical studies of the air-water interface of a water droplet show a wide distribution of strong electric fields at the surface that can make or break chemical bonds to accelerate chemical reactions over the bulk water phase.

## Introduction

Recent exciting work has shown that seemingly simple water droplets give rise to unexpected rate accelerations for organic reactions by factors of one to six orders of magnitude compared to the bulk liquid^1,2^. Understanding how water droplets promote reactive chemistry has the potential for exercising greater control of the microdroplet environment that would permit synthesizing new compounds^2–4^, materials^5^, and using electrosprayed droplets to accomplish chemical analysis^6^ and decontamination^7,8^, all in ways that aren’t currently possible or well-optimized under conventional bulk reaction conditions.

Presently we do not fully know what makes water droplets exceptional for accelerating reactions, and as such it is a current and highly active area of investigation. Factors that may contribute to the rate acceleration include concentration increases due to solvent evaporation^9^, partial solvation of reactants^2,10^, gas-phase channels^11,12^, changes in pH^13^, a localized dielectric constant that deviates from bulk^14^, and favorable entropy changes due to preferential orientations of the reactant molecules near the surface^15^. There is experimental evidence that the droplet enables or recruits a surface active species and/or the possibility that the droplet charge state is driving the accelerated reaction chemistry^16–18^. The exact identity of the surface active or charged species is unknown^19^ and even the surface pH is still a matter of debate^20^. Some recent work suggests that there are trace impurities^17,19^, the presence of salts^13^ or bicarbonate from dissolved carbon dioxide^21^, while others suggest that it originates from different affinities of H_3_O^+^ and OH^−^ to the surface medium surrounding the spherical droplet^22,23^.

But one of the primary and more fundamental hypotheses about the interfacial features of a microdroplet is the presence of strong electric fields that can align with chemically reactive bonds to accelerate reactions relative to the bulk phase^24^. In particular we and others have shown that good electric field ${{{{{\bf{E}}}}}}$ alignment with the reactive bonds will accelerate the reaction by lowering the transition state barrier, $\triangle {G}^{{{\dagger}} }$ or possibly raising the reactant state energy through bond activation of a breaking bond of interest^24–30^. Estimates of the required electric field strengths to lower the activation energy in either scenario range from tens of MV/cm for bond activation^18^ to several hundred MV/cm for making or breaking strong chemical bonds or to induce redox reactions^24,28,31,32^. However, quantifying electric fields at the air-water interface is not straightforward, either experimentally or theoretically, and very limited and/or conflicting evidence has not fully established the magnitude of the surface potential and related interfacial electric fields^33–37^.

In this work we have utilized a reactive force field model of water, ReaxFF/C-GeM^38,39^, that explicitly models coarse-grained electrons and thus the internal electronic charge distribution of the water molecule^40^, and yet can well describe the structural organization and dynamics of water for relatively large sub-micron droplets over tens to hundreds of nanoseconds, size and timescales that are not accessible with ab initio molecular dynamics (AIMD). Here we use the model to simulate the electric fields of large droplets of 80–160 Å in diameter to characterize their field strengths at the air-water surface, and to evaluate the electric fields for different charge states of the water droplets with an excess of Na^+^ ions, Cl^−^ ions, H_3_O^+^ ions, OH^−^ ions and Na^+^/Cl^−^ ion mixtures. We find that electric fields at the surface are larger and well-aligned with the free O–H groups at the surface relative to electric field alignment for arbitrary water bonds in the inner droplet. In particular we find field strengths that increase to an average  of ~16 MV/cm, enough to activate strong bonds or break weak chemical bonds, with a wide distribution of field strengths that can reach an order of magnitude larger to drive faster chemical reactions.

Our works shows that the nature of chemical reactivity at the air-water interface is sensitive to *both* structural organization and electronic organization at the interface^34^, with Lorentzian distributions of electric field strengths generated at the microdroplet surface that arise from non-linear coupling of intramolecular solvent polarization with intermolecular solvent dynamical modes. We further suggest that the broader electric field distributions we observe after projection onto the free O–H bonds at the surface could be evidence of how the large number of droplets generated in the electrospray process statistically sample with greater field alignment along reactant bonds than the average electric field, and further promoting reactive chemistry.

## Results

It has been well-corroborated both experimentally and theoretically that the topmost water layer of the air-water interface is organized with a majority of O–H dangling bonds pointing toward the vapor phase^41,42^. Just beneath the topmost layer and parallel to the instantaneous fluctuating surface there is a two-dimensional hydrogen bond network^43,44^ that supports the “free O–H” configuration. These structural and dynamical features of the air-water interface, which has a thickness of ~2 solvation layers deep^44^, will yield electric field signatures that are expected to be distinct from the bulk-like interior of the droplet. Indeed, the free O–H of the water molecules at the surface generates asymmetric stretching frequencies that are highly sensitive to their hydrogen-bonding structural and electronic environment and/or presence of excess ionic charge. As such, they are also direct reporters of surface electric fields as shown by Cooper et al. using Infrared Photodissociation (IRPD) spectroscopy. In this experiment, the measured Stark shifts are linearly proportional to the local electric field at the surface^45^, and depend on both cluster size and the absence or presence of ions and their identity^46^.

**Fig. 1:**
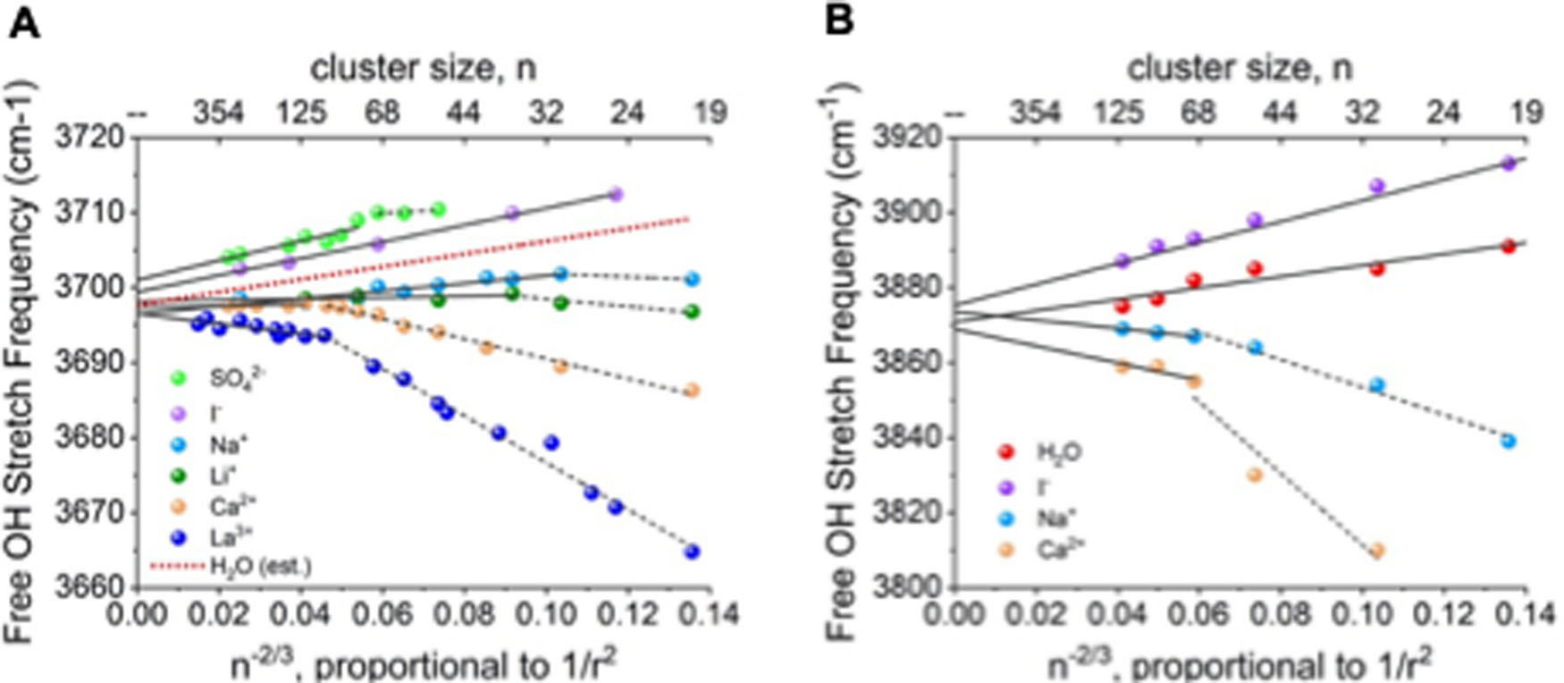
*Frequencies of the AAD free OH bands from spectra of (H*_*2*_*O)*_*n*_*and M(H*_*2*_*O)*_*n*_. Here we compared experiments and theory for M = Ca^2+^, Na^+^, I^−^ and Cl^−^ as a function of n^−2/3^, which is proportional to 1/r^2^ where r is the droplet radius. **A** Experimental results from IRPD spectroscopy; adapted with permission from the Royal Society of Chemistry^46^. **B** Results using the ReaxFF/CGeM force field.

Figure 1A displays the experimental IRPD measurement which shows that the neutral water and anionic water clusters exhibit a red-shift of the free O–H band with increasing cluster size^46–48^, whereas for positively charged cations there is a blue-shift of the free O–H band when progressing to larger clusters, accompanied by a transition in slope that correlates with the ion’s charge (*n* = 100 and 30 for Ca^2+^ and Na^+^, respectively). This trend is not reproduced using simple fixed charge models that instead exhibits a linear function of the Stark shift with 1/r^2^ (Supplementary Fig. 1), and is an important indicator that the surface features of microdroplets arise from many-body effects such as charge transfer^40,49^ and intramolecular and intermolecular polarization^50–52^. Figure 1B verifies that the Stark shift trends are very well captured by the ReaxFF/C-GeM model (although our frequency range is too high compared to experiment), which is relevant for not only the validation of the simulation model, but plays an important interpretative role in analyzing the electric field for large water droplets in terms of electron density, protonation states, and ion effects at the air-water interface.

**Fig. 2:**
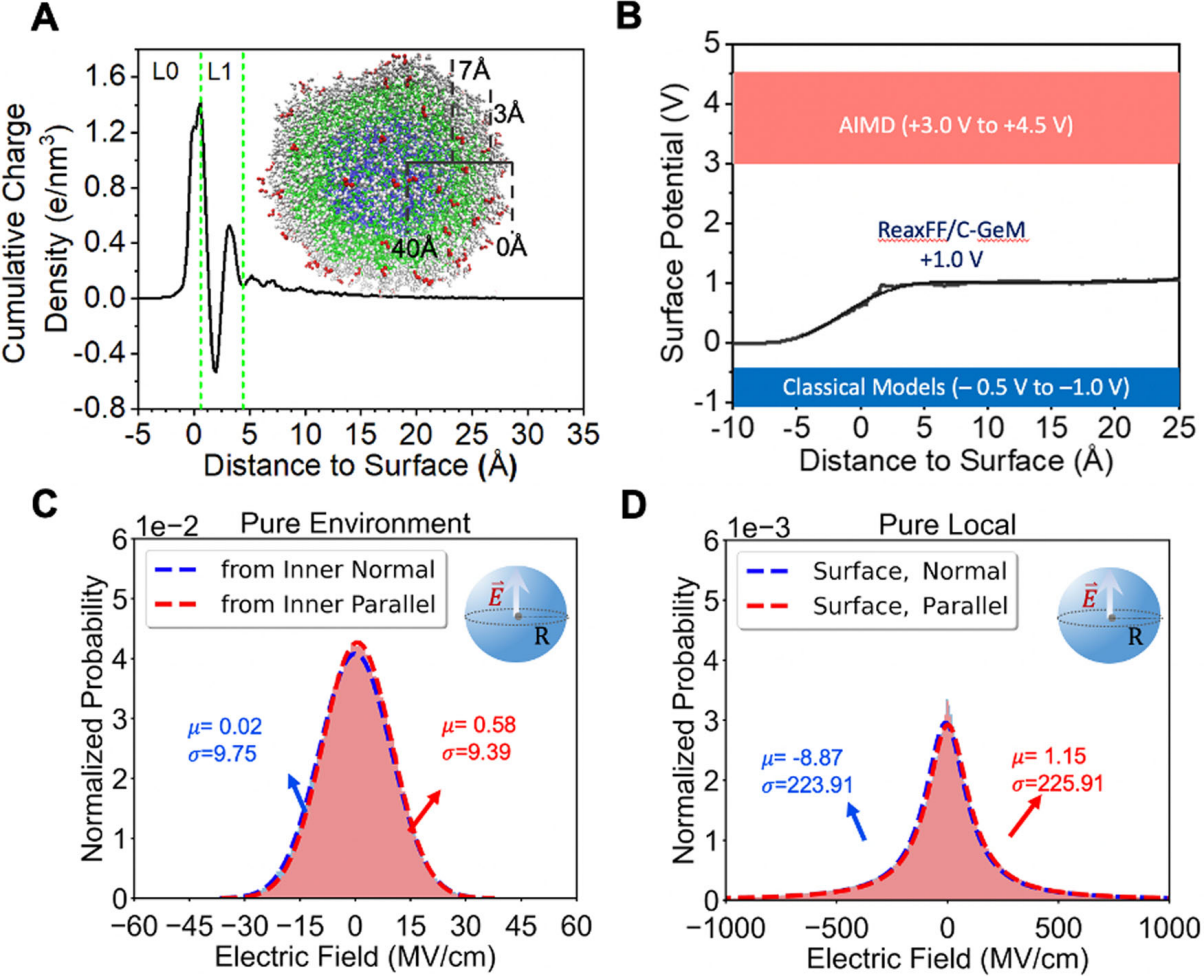
*Interfacial electric fields for pure water droplets*. **A** Cumulative charge density using the ReaxFF/C-GeM model. The inset shows a cross-section of the droplet with a 40 Å radius with the surface waters depicted in gray as measured for the instantaneous surface. The green dotted lines pertain to definitions of L0 and L1 regions. **B** The variations in surface potential by radial regions in the R40 water droplet. **C** Inner water droplet contributions to the electric field at the surface are negligible and show a Gaussian distribution. **D** Electric field distributions at the surface are seen to be Lorentzian as measured over L0-L1. The electric field is largest in the normal direction to the interface. Here we use a grid resolution of 1.0 Å, although the results are the same with a finer 0.25 Å resolution (see Supplementary Fig. 4A).

Now we turn to much larger droplets of 40–80 Å in radius (R40–R80)—large enough so that curvature effects are negligible—to evaluate the electric field contributions from the inner droplet and at the surface. The neutral water droplet charge integrates to zero and thus it obeys Gauss’s law as shown in Fig. 2A, and further details are provided in Supplementary Fig. 2 in regards the electric field calculations that further establish expected theoretical limits. As seen in Fig. 2B the surface potential determined with ReaxFF/C-GeM is positive like AIMD but with a smaller magnitude of ~+1.0 eV. ReaxFF/C-GeM is qualitatively different than classical force fields^35^ that yield a negative surface potential, because it can represent the “mean inner potential” arising from the  charge density of the interiors of water molecules^34^. To verify this, Supplementary Tables 1 and 2 show the electric fields inside the water molecules are very similar between ReaxFF/C-GeM and DFT (using the B97M-rV functional^53,54^ with the TZV2P basis set in CP2k^55^), and any differences in the interior electron density between the two are no worse than variations among DFT functionals as reported by Medvedev et al.^56^.

However the physical air-water interface, which has molecular granularity and an asymmetric and heterogeneous charge distribution, fluctuates as seen in Fig. 2A. In particular, the accumulated charge density increases in the L0 region to positive charge values, then decreasing in the L1 region to net negative charge density, before increasing rapidly again to positive charge density. This behavior arises from the electronic shells that are displaced such that the atomic cores are less shielded at the outermost L0 surface, and resulting in an increase in the electronic density in the L1 region, and smoothing out to zero within the droplet. This helps to distinguish the surface from the droplet interior, so that we can analyze the electric fields by region in the normal ${{{{{{\boldsymbol{E}}}}}}}_{{{\perp }}}$ and parallel ${{{{{{\boldsymbol{E}}}}}}}_{{{\parallel }}}$ directions to the surface. Figure 2C, D confirm the expectation that the $\left\langle {{{{{{\boldsymbol{E}}}}}}}_{{{\parallel }}}\right\rangle$ contributions are zero. Figure 2C shows that the “environmental” electric field contributions from the inner droplet have no net orientational effects to contribute to a surface enhanced electric field, and are Gaussian distributed with relatively small variance.

But locally at the surface the electric fields exhibit an orientational preference for a surface normal as seen in Fig. 2D, with a mean value of −8.9–9.2 MV/cm when averaged over electric fields evaluated directly from grid points, or yielding −12.0 MV/cm if taking the slope of the surface potential in Fig. 2B. Both estimates from two independent calculations are in good agreement with each other and in excellent agreement with a recent Stark analysis of ~10 MV/cm measured by Stimulated Raman Excited Fluorescence (SREF) spectroscopy^36^. We note that when we use DFT with its small system size and a slab geometry (because a R40 droplet is not affordable with AIMD), the derivative of the surface potential gives ~150 MV/cm, but the electric field sampled over a grid yields a value that is much smaller, ~50 MV/cm (see Supplementary Fig. 5). We believe that this numerical evidence indicates there is a problem of a finite size effect in the QM calculations, i.e., the DFT result doesn’t show consistency between the direct electric field calculation and the derivative of the surface potential.

Nonetheless, Fig. 2D also shows that the electric fields at the surface exhibit a non-Gaussian distribution, with a large variance in field strengths of hundreds of MV/cm. This is a consequence of the non-linear coupling of the intramolecular polarization of a water molecule with the intermolecular solvent modes as anticipated by Matyushov and Voth^50^, as well as representing the large electric fields arising from the sampling of the inner potential of water molecules. What we learn from Fig. 2 is that there is a very localized orientational preference for the surface normal for electric fields whose magnitude is consistent with experimental SREF measurements, but with strong heterogeneity such that electric fields can be ~30X larger than the average which is a magnitude consistent with previous experiments^37^ and ab initio studies^33,34^.

**Fig. 3:**
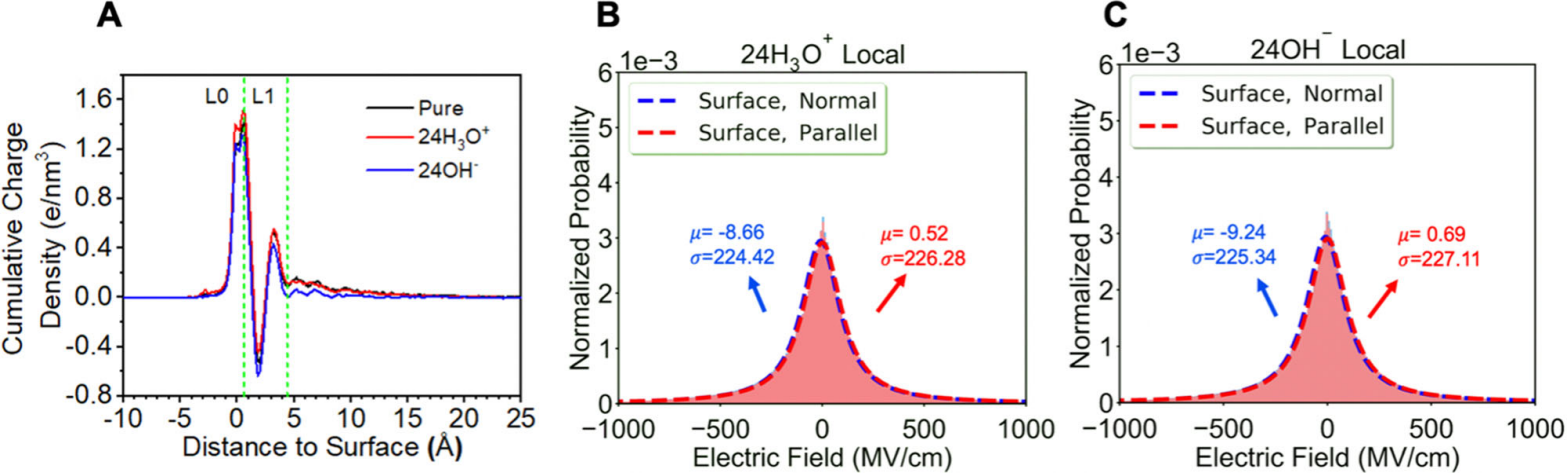
*Interfacial electric fields of droplets in the presence of excess hydronium and hydroxide ions*. **A** Cumulative charge density for pure water and in presence of excess H_3_O^+^ or OH^−^. The Lorentzian signatures of the electric fields normal to the surface for the R40 water droplet with (**B**) H_3_O^+^ and (**C**) OH^−^ corresponding to 88% of the Raleigh limit.

In regards an additional surface-active species, we consider an excess of cationic species in the form of 24 H_3_O^+^, or an excess of anions using 24 OH^−^ (~88% of the Rayleigh limit for the R40 droplet) as given in Fig. 3. The ion distribution profiles are provided in Supplementary Fig. 6, and show that H_3_O^+^ ions have a greater propensity for the surface while the OH^−^ ions are better mixed and are distributed throughout the surface and inner droplet region. With an excess of 24 H_3_O^+^ the small charge density increases seen in Fig. 3A are consistent with the larger Stark shifts seen with small nanodroplets in the presence of cations, whereby electron density displaces toward the hydronium charge and deshields the hydrogen of the free O–H at the surface to create an even larger positive surface potential compared to pure water. By contrast an excess of OH^−^ anions push greater electron density onto the exposed O–H bond at the surface such that the magnitude of the Stark shift is found to be smaller due to a reduction of the positive surface potential. But the interplay between polarization effects whereby the coarse-grained electrons organize differently in the L0 vs. L1 region are compensated by structural variations of the nuclear centers as well, yielding very little difference in the integrated electric field profiles. At best local electric fields introduced by the ions slightly shifts the average to slightly lower electric field average for H_3_O^+^ ions (Fig. 3B) and slightly higher electric fields for OH^−^ (Fig. 3C). Similar trends are observed for excess Na^+^ and Cl^−^, or corresponding salt mixtures (Supplementary Table 3 and Supplementary Fig. 7). While the electric field shifts we observe in the presence of ions with respect to bulk water are small due to electrostatic screening, closer to the ions the electric fields can be quite large. In addition we also note that the effective concentration of ions is still quite small and a proper modeling of the electrospray process in regards charge fragmentation is warranted in future work.

**Fig. 4:**
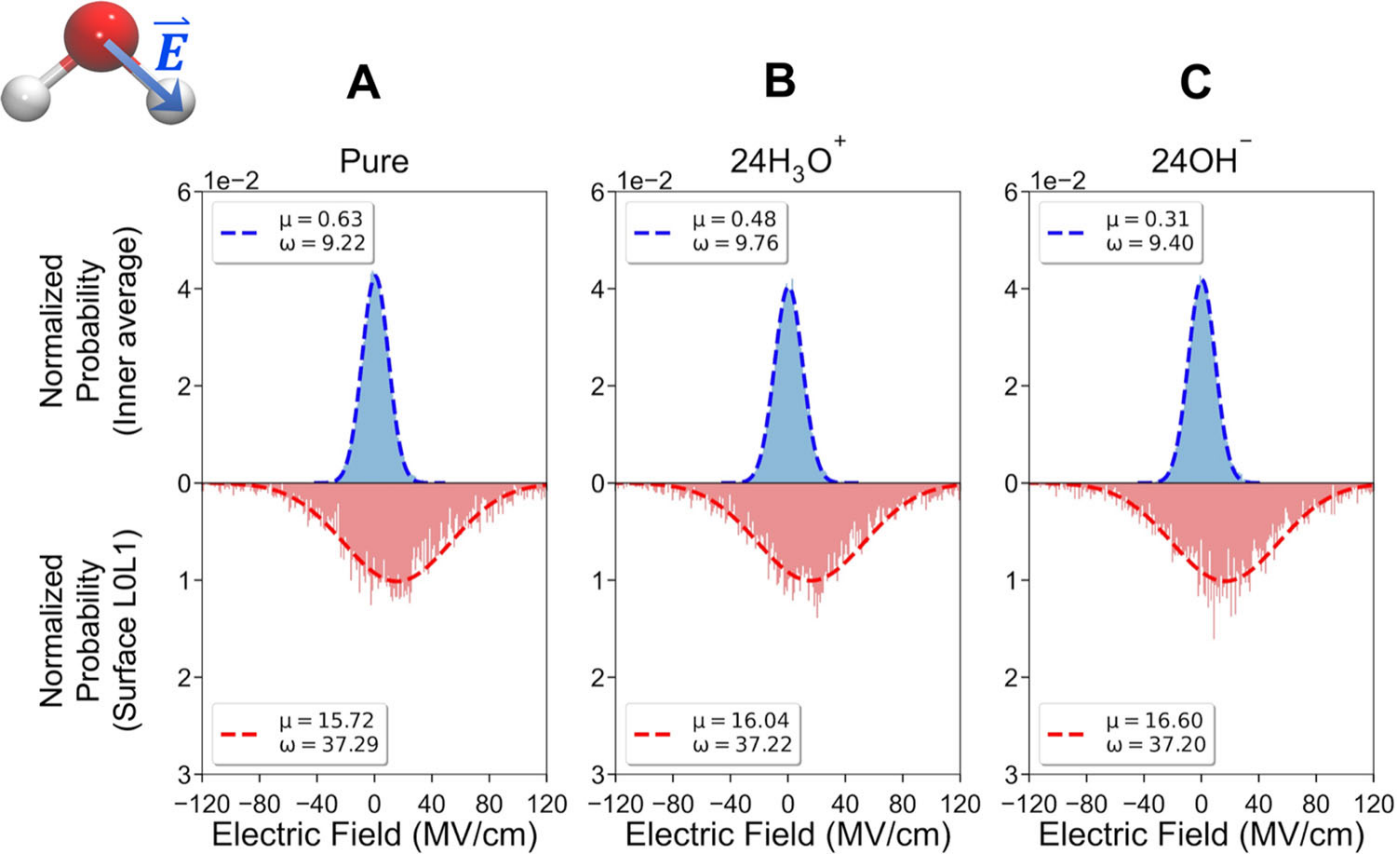
*Electric field distributions arising from electric field projections on the O–H bonds of water*. The hydrogen-bonded water molecules in the inner droplet region (blue) and the surface free O–H water bonds (red) for the R40 droplet with (**A**) pure water, water with (**B**) 24 H_3_O^+^ and (**C**) 24 OH^−^ ions.

To further analyze the effect of the electric field that can potentially catalyze chemical reactions and break chemical bonds in a microdroplet, we project the electric fields onto the O–H bond vector of water molecules in the inner droplet and onto the free O–H bond at the surface (Fig. 4, and Supplementary Fig. 8 for the sodium and chloride ions). In this case the electric field grid points within 1 Å of any atom of the molecule onto which we project is eliminated from the electric field in order to measure the external electric field on the chemical reactivity. For a pure water droplet, or in the presence of charge, the surface free O–H bonds are more destabilized due to an average projected electric field of an ~16 MV/cm compared to the interior droplet water molecules.

The significance of ~16 MV/cm increase in field strengths when projected onto bonds at the air-water surface would correspond to lowering an energy barrier, and the impact on chemical transformations would depend on the type of bond being broken and whether they are close to ions if they are present. The transition state lowering can be estimated in a number of ways^30^, but here we consider a simple bond dipole-field model^57,58^

1 $${\triangle {G}}^{{{\dagger}} }=-\mathop{\sum}\limits _{{reactive}\atop{bonds}}{{{{{\mathbf{\mu }}}}}}_{{{TS}}^{{{\dagger}}}}\cdot ({{{{{\bf{E}}}}}}_{surface}\;{{{{{\boldsymbol{-}}}}}}\;{{{{{\bf{E}}}}}}_{interior})$$

for breaking a water bond. We estimate the bond dipole in the transition state to be 2.75 D (~25% larger than the 2.2 D in the ground state) yielding a free energy lowering of ~2.1 kcal/mol, which in the exponential would increase the equilibrium constant of water or the rate of reaction by 1–2 orders of magnitude. The much wider wings of the Gaussian as seen in the electric field projections on surface bonds in Fig. 4 potentially increases the odds for chemical bond weakening or dissociation relative to bulk water, which may be relevant in the electrospray process that generates a large number of droplets of various charge states.

## Discussion

Electric fields are highly sensitive to both the nuclear arrangements of water molecules at the surface as well as the electron density response at the air-water interface that may be a vital clue to understanding the reactive chemistry of microdroplets. The surface potential and its derivative property, i.e., electric fields, at the air-water interface has been difficult to reconcile by experiment and theory. Some of the issues experimentally are challenges of sensitivity to and spatial resolution at the interface, the choice of appropriate optical or spectroscopic probes, and differences in whether or not the measurements also sense the internal electronic distribution of the water molecules^33–35^. Theoretically, Leung^33^, Kathmann^34^, and later Cendagorta and Ichiye^35^ have highlighted the different assumptions made by ab initio methods vs. classical force fields in the evaluation of surface potentials and electric fields and the experiments used to validate them. Point-charge models only probe the charge density outside of the water molecules, and represents what Kathmann calls the “electrochemical potential”, because the probe is excluded from the interior of the water molecules and thus measures lower surface potentials of (typically) negative sign. AIMD using Density Functional Theory also has contributions from the “mean inner potential” by averaging over all space that includes the electron density of the interiors of the water molecules, and yields higher interfacial electric field strengths of positive sign^33,34^. The AIMD results are consistent with electron holography experiments that sense the electronic density within the solvent molecules^37^, and thus appear to more heavily weight the mean inner potential, unlike classical force fields that don’t have a contribution from the mean inner potential at all. The ReaxFF/C-GeM yields a positive surface potential like AIMD because it also represents the “mean inner potential” due to charge density of the interiors of water molecules with an error no worse than found by variations in DFT functional^56^.

However the AIMD system sizes are too small and thus show inconsistency in the electric fields calculated on a grid vs. taking the derivative of the surface potential, whereas the ReaxFF/C-GeM model^38^ yields consistent average surface electric field values of approximately −10.0 MV/cm by both methods. Our results are in excellent accord with the electric field magnitudes from SREF experiments of neutral droplets^36^ and show good accord with infrared measurements at the surface of small charged water clusters containing simple inorganic ions^46^. Because the mean inner potential is important, it manifests as a Lorentzian distribution of electric field strengths due to non-linear coupling of intramolecular polarization with intermolecular solvent modes that represent these higher electric fields. We further suggest that the broader electric field distributions we observe after projection onto the free O–H bonds at the surface could be evidence of how the large number of droplets generated in the electrospray process statistically enhance reactivity through reactant bonds with greater alignment than the average electric field.

While a recent study concerning the surface charge at the air-water interface has determined a small negative surface potential^49^, in disagreement with ab initio calculations and ReaxFF/C-GeM, what we believe is mutually supportive with this previous work is how both the structure and electron density is organized at the interface^22,59,60^. It is a critical factor for estimating surface charge^49^, surface potentials, electric field strengths, and electric field projections important for surface chemical reactivity which is the topic here. Because there is a strong normal orientational preference for electric fields that are very localized at the air-water interface, i.e., a type of electrostatic pre-organization, it can provide an explanation for the lowered barrier of chemical reactions in microdroplets. We estimate from a bond dipole-field model using a water molecule “reactant” a lowering of the transition state energy for bond breaking with a ~3kT effect on average. As is already known, all microdroplet accelerations are modest relative to a highly optimized catalyst, but at the same time there is a strong heterogeneity such that electric fields can be ~30X larger than the average electric field and indicates that rate accelerations can be much higher depending on fluctuations and sheer numbers of droplets generated in the electrospray process. In summary, this work confirms the importance of surface electric fields as a source of microdroplet reactivity that should be investigated for the range of organic reactions in which accelerations are observed^1–3,7,9,13^, and relevant controls where the microdroplet may not always reach greater reactivity rates relative to the bulk water liquid^10,61^.

## Methods

Each system was minimized and equilibrated using the AMOEBA force field within the Tinker-OpenMM platform^62^. The system was heated in the NVT ensemble from 50 to 300 K at a rate of 0.33 K/ps using a Bussi thermostat and RESPA integrator with a 1 fs timestep^63^. Once the systems reached 300 K, another 3 ns simulation was run in the NVT ensemble for equilibration. Ewald cutoffs of 9 Å and van-der Waals cutoff of 12 Å were used. The cubic box was set to be (120 Å)^3^, (160 Å)^3^, and (200 Å)^3^ for R40, R60, and R80 droplets, respectively. The systems were then transferred into a recent implementation in LAMMPS^39^, where the reactive force field ReaxFF/CGeM model has been implemented and the MD trajectories were conducted. After 500 ps of equilibration, we collected snapshots every 1 ps across a 400 ps to 1 ns production run to obtain the electric field.

### IRPD calculations

For the IRPD studies, the *n* = 70 initial structures were taken from a recent study by Paesani et al.^64^, and the structures for *n* = 20, 30, and 50 were extracted from the *n* = 70 cluster. For the electric field calculations, the initial configurations of the water droplets with radius ranging from 40 Å (8600 water molecules) to 80 Å (71,000 water molecules), with and without ions, were first prepared using the PACKMOL^65^ software package.

### Ion concentrations

The formed evaporating charged droplets will quickly reach a point, known as the “Rayleigh limit”, after which they are no longer mechanically stable^56^. This condition is known as the Rayleigh instability, which is the maximum number of surface charges, Q_R_, that can exist on a droplet of radius, R_R_, when electrostatic repulsion is balanced by surface tension. This is given by Eq. (2), where ${\epsilon }_{0}$ is the permittivity of free space and $\gamma$ is the liquid surface tension:

2 $${Q}_{R}=8\,\pi {\left({\epsilon }_{0}\gamma {R}_{R}^{3}\right)}^{1/2}$$

At T = 300 K, the value of surface tension is taken to be 0.0523 N/m. We used Eq. (2) to determine ion concentrations in Fig. 3.

### Charge density profiles

In order to determine the charge density profile, we considered the instantaneous surface method^66^ to find the instantaneous interface, and further defined L0 and L1 where charge density varied by distance in Figs. 2A and 3A. For the cumulative charge density calculation *only* we collapsed the Gaussian densities to point charge centers (+1 for cores and −1 for shells) starting from the outer of air-water interface to the droplet center. The accumulated charge density is the averaged in the volume from the outside the air-water interface (here we defined it as −5 Å where the integrated charge density is found to be zero) and is calculated every 0.2 Å throughout the distance scan.

### Surface potential and electric fields on a grid

The surface potential is defined as

3 $${V}_{{ij}}^{{surface}}\left({r}_{{ij}}\right)=\frac{{q}_{i}}{{r}_{{ij}}}{erf}\left(\sqrt{\frac{{\alpha }_{i}{\alpha }_{j}}{{\alpha }_{i}+{\alpha }_{j}}}{r}_{{ij}}\right)$$

where ${i}$ denotes the Gaussian core and shell position, ${q}_{i}$ is the Gaussian density at a grid point $j$ at which a test charge ${q}_{j}$ = +1 is placed to evaluate the potential; we use a high value of ${\alpha }_{j}$ to approximate the point charge and ${r}_{{ij}}$ is the distance between the grid point and the Gaussian core or shell. The electric field, which is the derivative of the electrostatic potential as implemented in LAMMPS, is done accordingly on the same grid points. We averaged the potential every 1 Å over 200 snapshots of production trajectories. We used Eq. (3) to determine Fig. 2B.

### Electric field projections

To obtain the electric field normal to surface, we project the electric field in the normal to surface direction

4 $${E}_{\perp }=\frac{{E}_{x}\cdot \left(x-{x}_{c}\right)+{E}_{y}\cdot \left(y-{y}_{c}\right)+{E}_{z}\cdot \left(z-{z}_{c}\right)}{\sqrt{\left[{\left(x-{x}_{c}\right)}^{2}+{\left(y-{y}_{c}\right)}^{2}+{\left(z-{z}_{c}\right)}^{2}\right]}}$$

where (${x}_{c}$, ${y}_{c}$, ${z}_{c}$) is the droplet center. We used Eq. (4) to determine normal to surface field in Fig. 2 and 3. 

The electric field projected on the O–H bond is done by averaging over all the grid points with 1/r^2^ factor, where *r* is the distance between the midpoint of the O–H bond and any given grid point. The grid points within 1 Å from the O–H bond midpoint were excluded to minimize the intra-molecular interaction effect from the interior of the water molecule. We used Eq. (5) to determine Fig. 4

5 $${E}_{O-H}=\frac{\mathop{\sum}\limits_{i}\left(\frac{1}{{r}_{i}^{2}}\right)\frac{{E}_{x,i}\;\cdot \;\left({x}_{H}\;-\;{x}_{O}\right)\;+\;{E}_{y,i}\;\cdot\; \left({y}_{H}\,-\,{y}_{O}\right)\;+\;{E}_{z,i}\;\cdot\; \left({z}_{H}\;-\;{z}_{O}\right)}{\sqrt{\left[{\left({x}_{H}\;-\;{x}_{O}\right)}^{2}\;+\;{\left({y}_{H}\;-\;{y}_{O}\right)}^{2}\;+\;{\left({z}_{H}\;-\;{z}_{O}\right)}^{2}\right]}}}{\mathop{\sum}\limits_{i}\left(\frac{1}{{r}_{i}^{2}}\right)} $$

## Supplementary information

Supplementary Information

## Data Availability

The datasets generated during and/or analyzed during the current study are available from the corresponding author on reasonable request.

## Code availability

The in-house scripts used to generate all data in the paper are organized in a private github, but will be made available from the corresponding author on reasonable request.
