# Supplementary material for: Can electric fields drive chemistry for an aqueous microdroplet?
Source: Nat Commun. 2022 Jan 12;13:280. doi: 10.1038/s41467-021-27941-x (PMC8755715; doi:10.1038/s41467-021-27941-x)
Supplement: Supplementary file 1 — Supplementary Information [file 41467_2021_27941_MOESM1_ESM.docx]

**Can Electric Fields Drive Chemistry for an Aqueous Microdroplet?**

H. Hao et al

**Supplementary Information**

**Can Electric Fields Drive Chemistry for an Aqueous Microdroplet?**

Hongxia Hao^1-3^, Itai Leven^1-3^, Teresa Head-Gordon^1-5^

*^1^Chemical Sciences Division, Lawrence Berkeley National Laboratory*

*^2^Pitzer Center for Theoretical Chemistry and Departments of Chemistry^3^, Bioengineering^4^ and Chemical and Biomolecular Engineering^5^, University of California, Berkeley, California 94720*


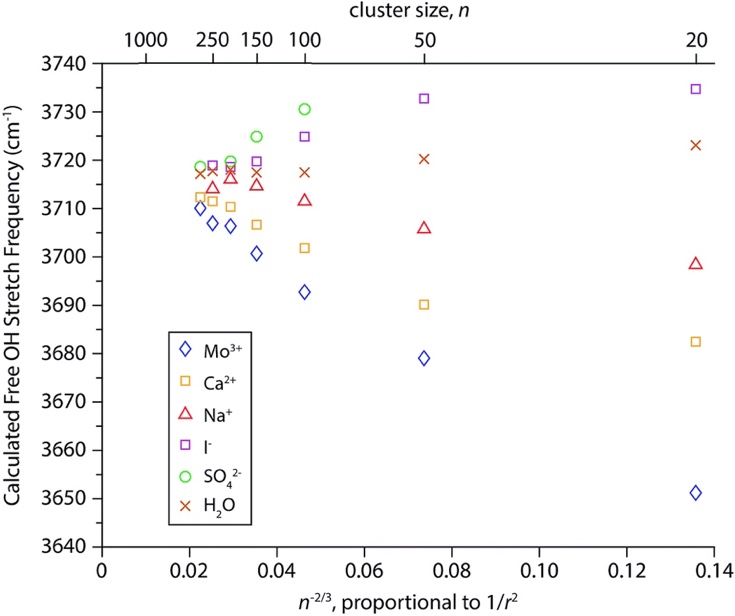


**Supplementary Figure 1.** *Centroid frequencies of the AAD free OH bands from simulated spectra using an electrostatic point charge model.* Frequencies of the AAD free OH bands from spectra of (H_2_O)_n_ and M(H_2_O)_n_. Here M = Mo^3+^, Ca^2+^, Na^+^, I^–^ and SO_4_^–2^ as a function of n^-2/3^, which is proportional to 1/r^2^ where r is the droplet radius. Simulation with the OPLS-2005 force field; adapted with permission from the Royal Society of Chemistry.^1^

**A B**


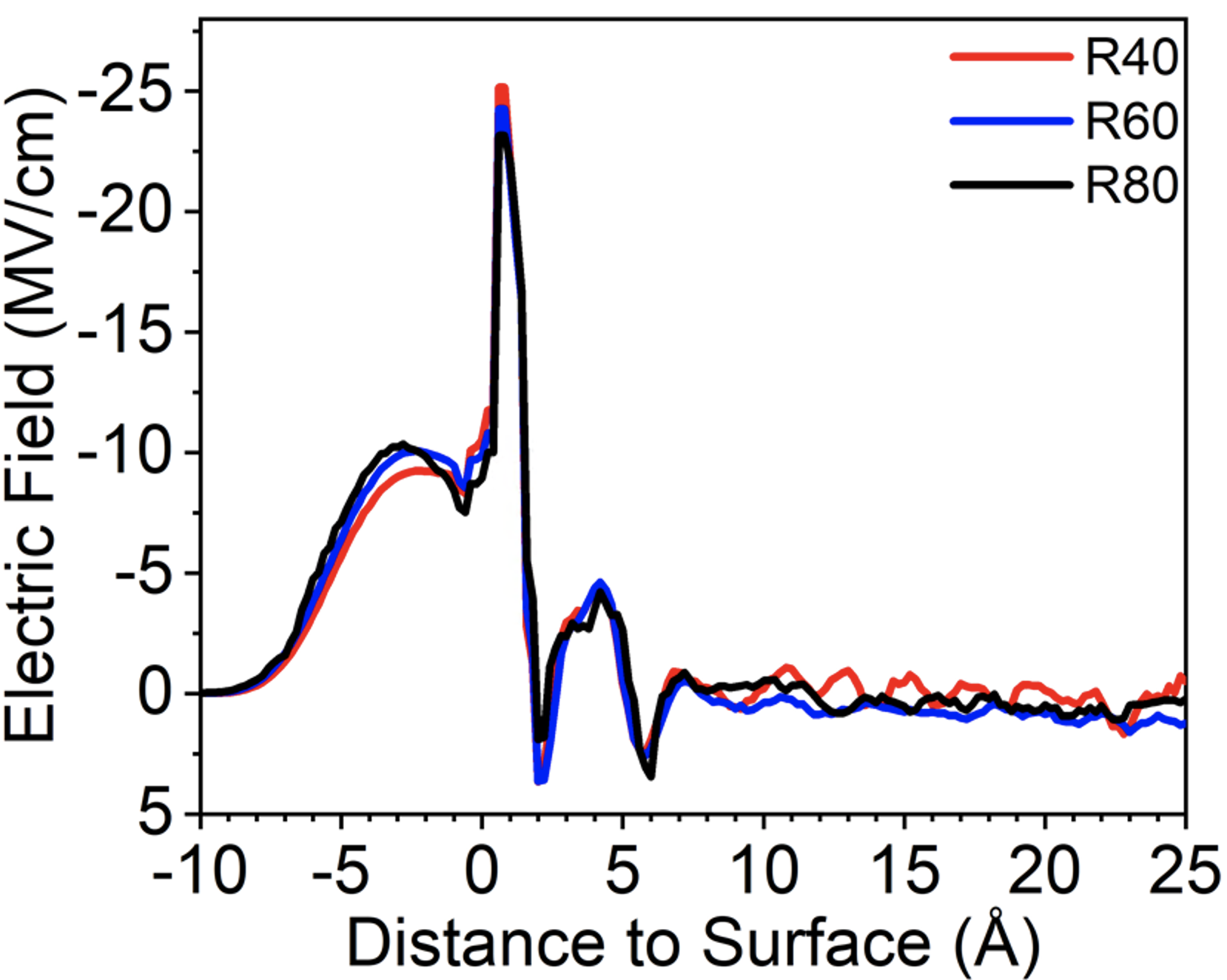

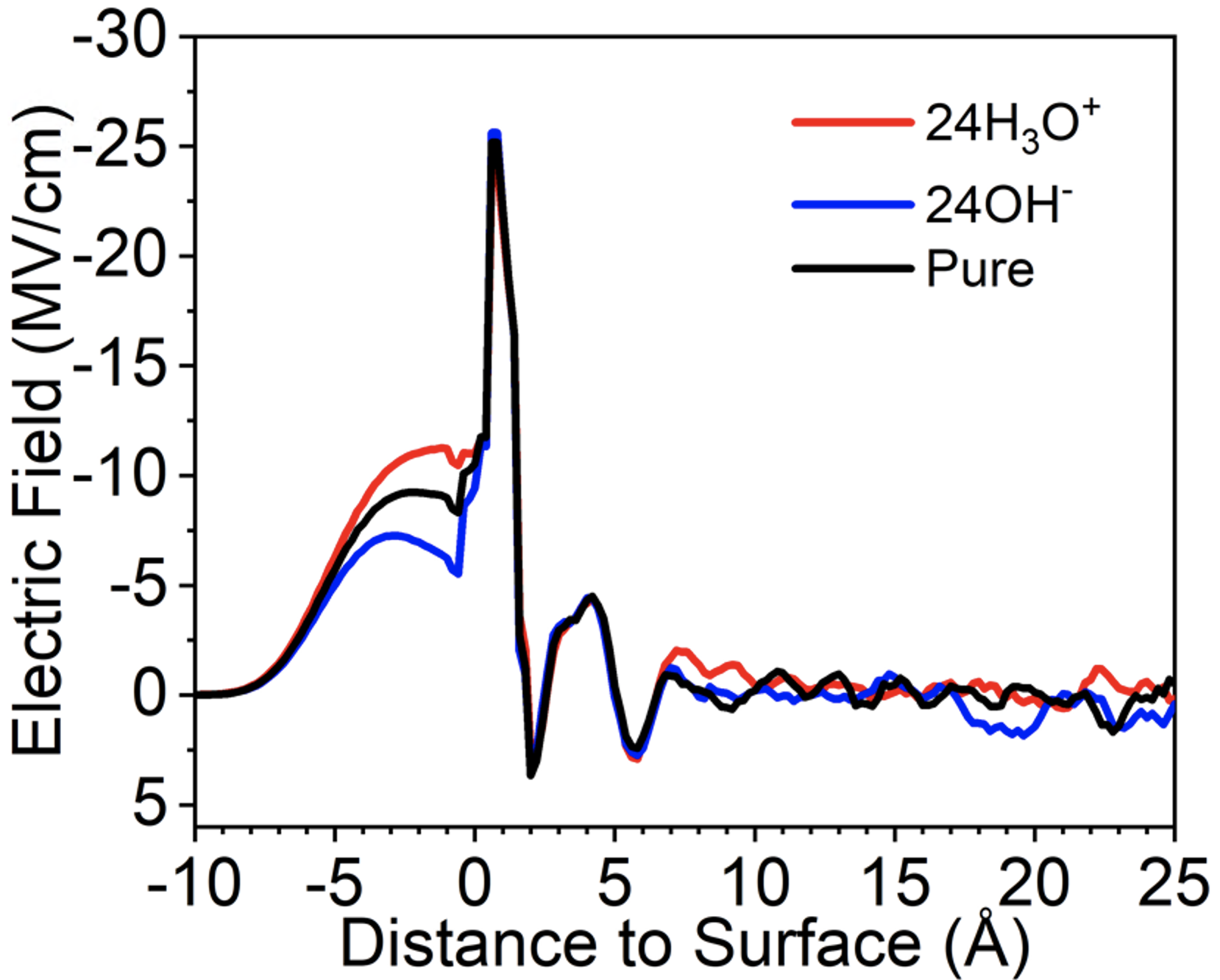


**Supplementary Figure 2.** *Evaluation of normal to surface electric fields using the ReaxFF/C-GeM model to show that Gauss’s law is satisfied*. (A) Pure water droplet as a function of size and (B) R40 droplet as a function of charge. The electric field goes to zero with a different distance dependence than cumulative charge in Figure 2A, but nonetheless confirms that Gauss’s law is satisfied. Figure 2A and **Supplementary Figure 2** correlate quite well, but the numerical issue of the last positive potential peak near 6 Å is a difference in calculation between cumulative charge density (which replaces Gaussians with point charges of core and shell to ease the calculation) vs. averaging over grid points with the full Gaussian density here.

**A B**


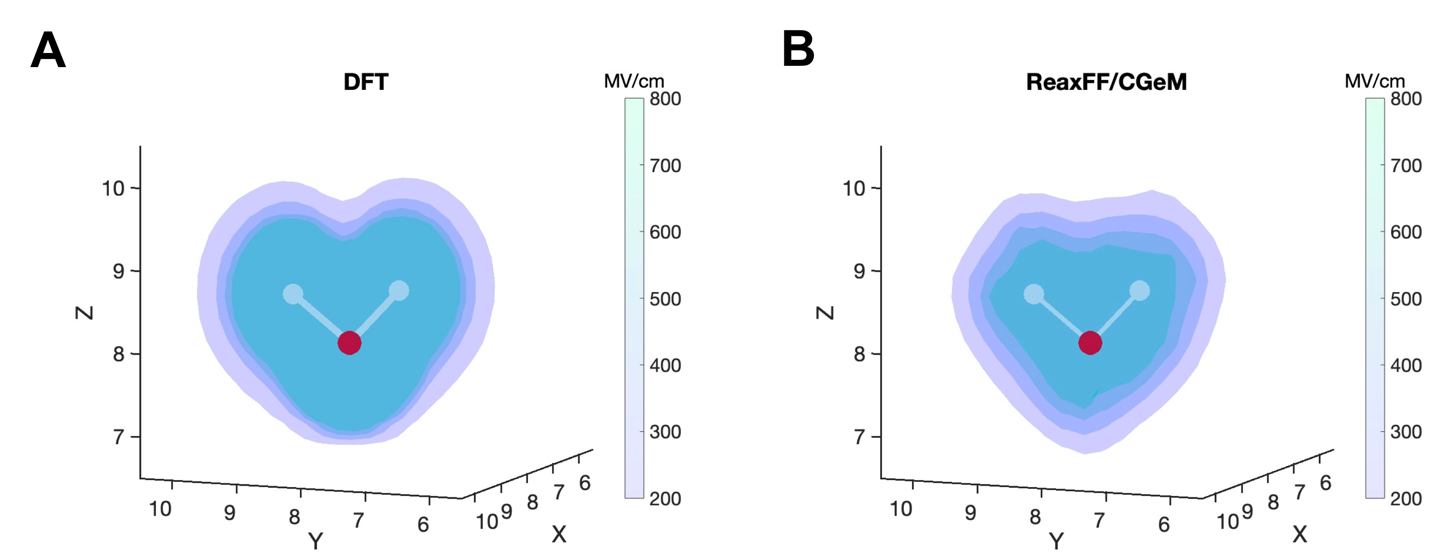


**Supplementary Figure 3.** *Evaluation of electric fields inside a single water molecule for (A) DFT using B97M-rV and (B) ReaxFF/C-GeM.* Evaluated using isovalues of 800, 600, 400, and 200 MV/cm with a grid spacing of (A) 0.26Å and (B) 0.25 Å respectively.

**A B C**


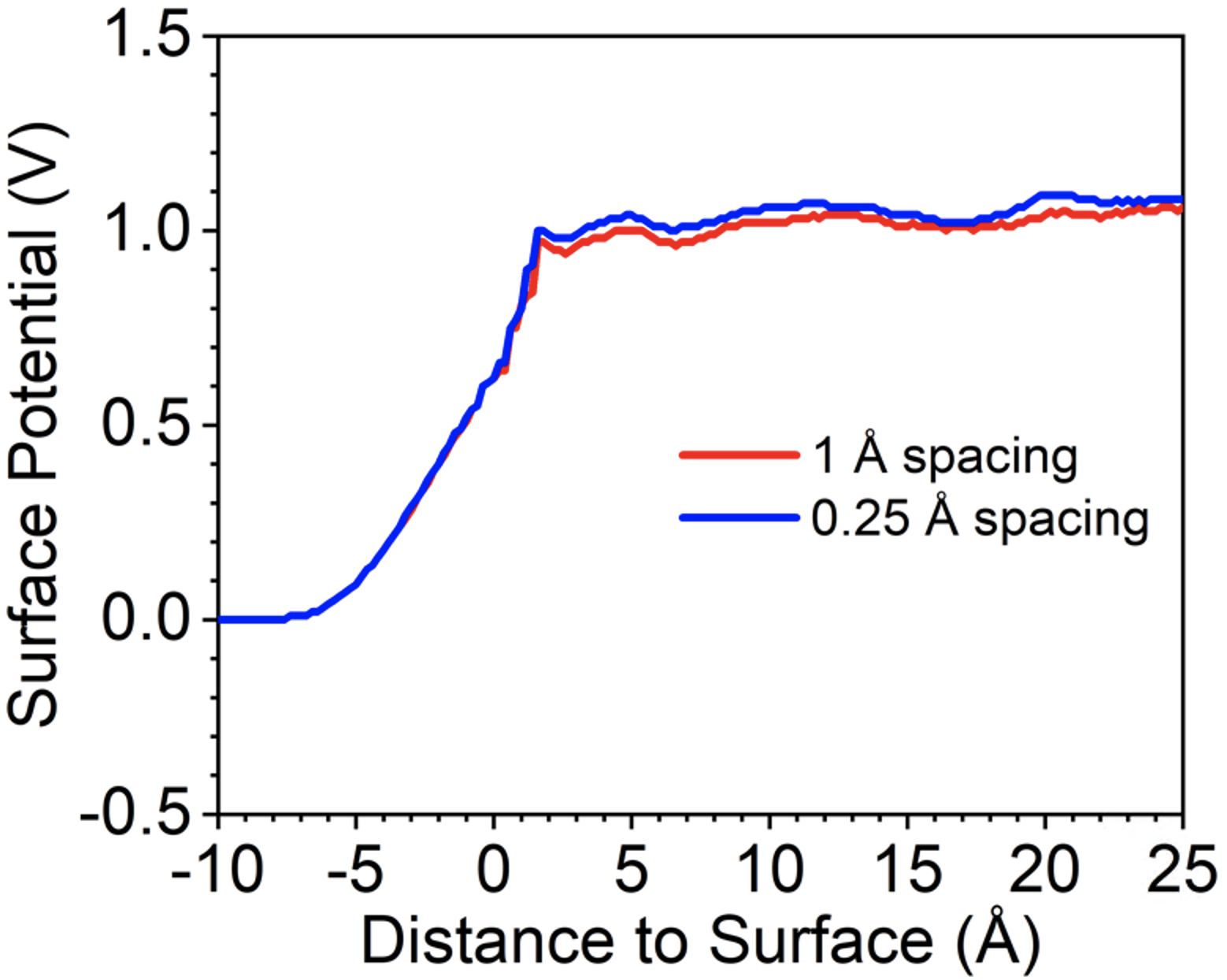

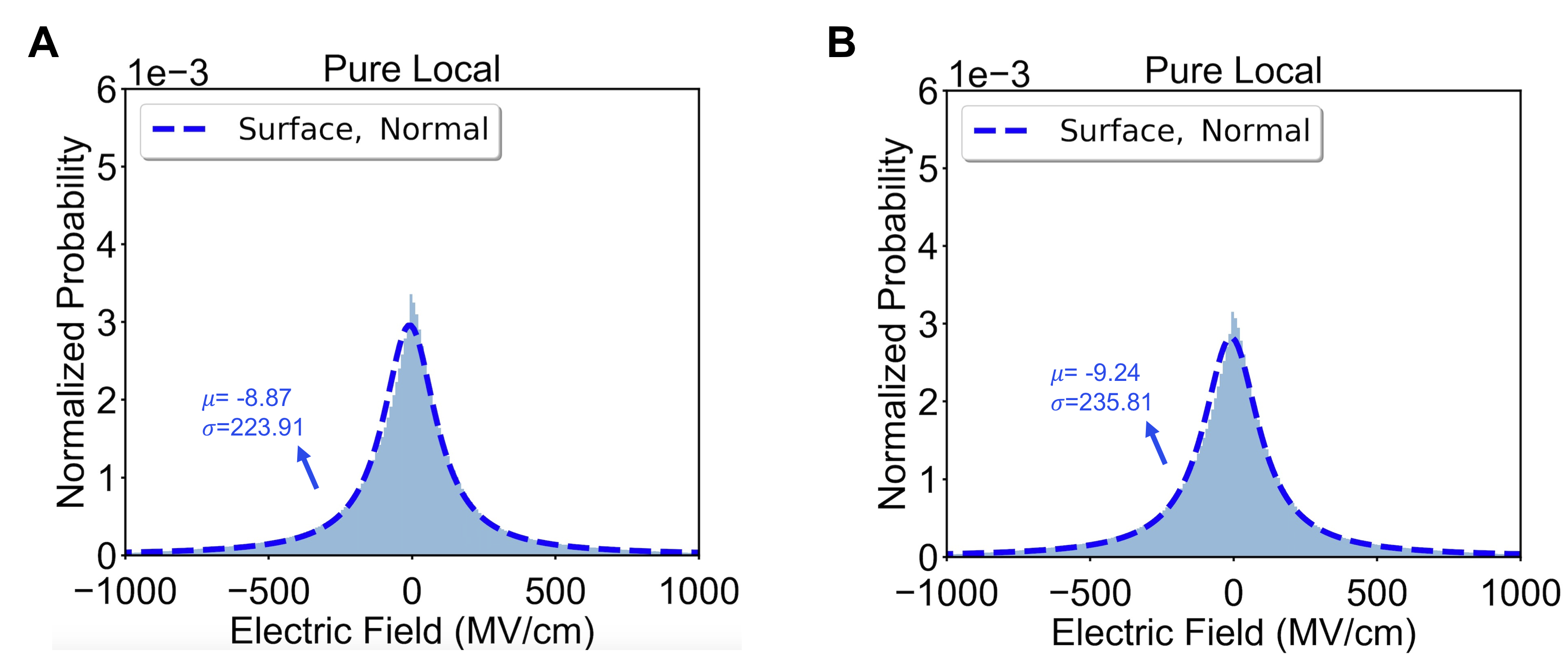

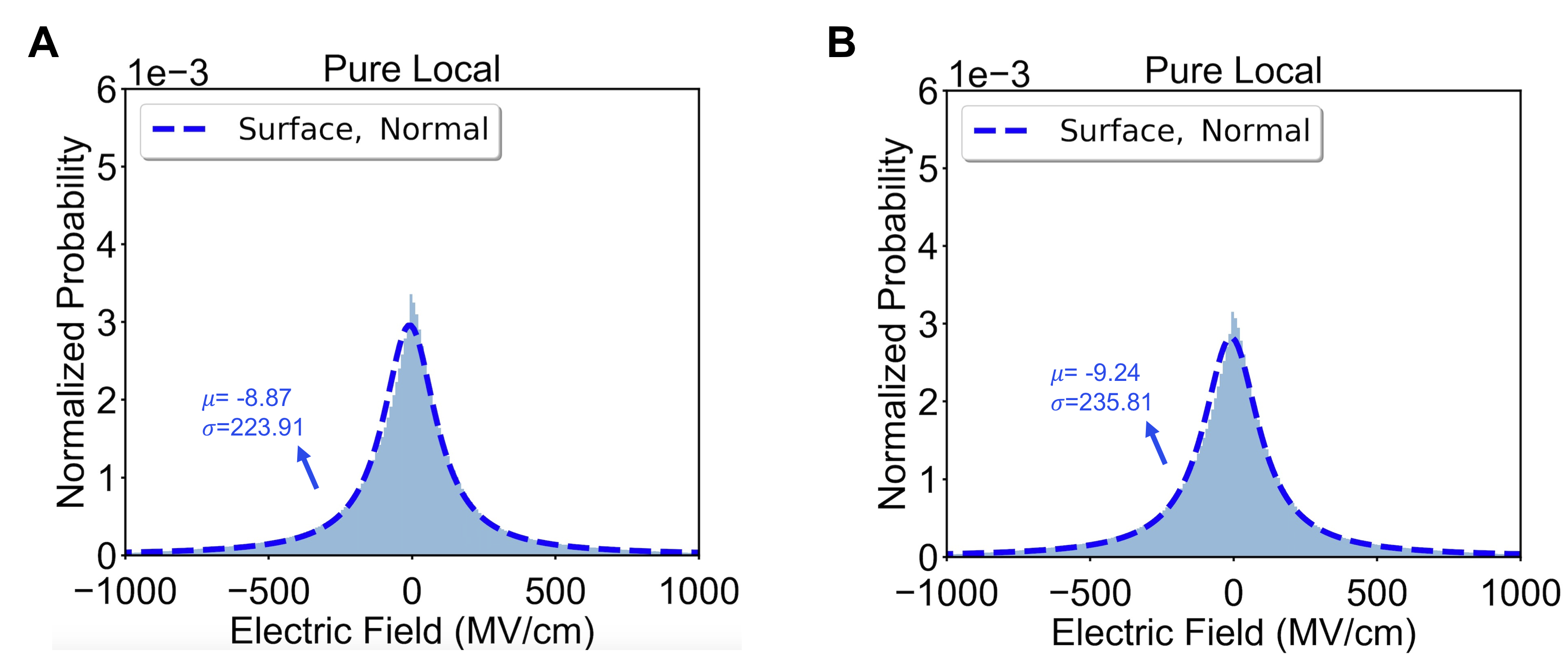


**Supplementary Figure 4.** *Evaluation of the surface potential and electric fields for the R40 water droplet using different grid spacings for the ReaxFF/C-GeM model.* (A) Surface potential at 1Å and 0.25Å spacing. Electric fields in the normal to surface direction for the R40 water droplet using (B) 1Å and (C) 0.25Å spacing.

**A B**


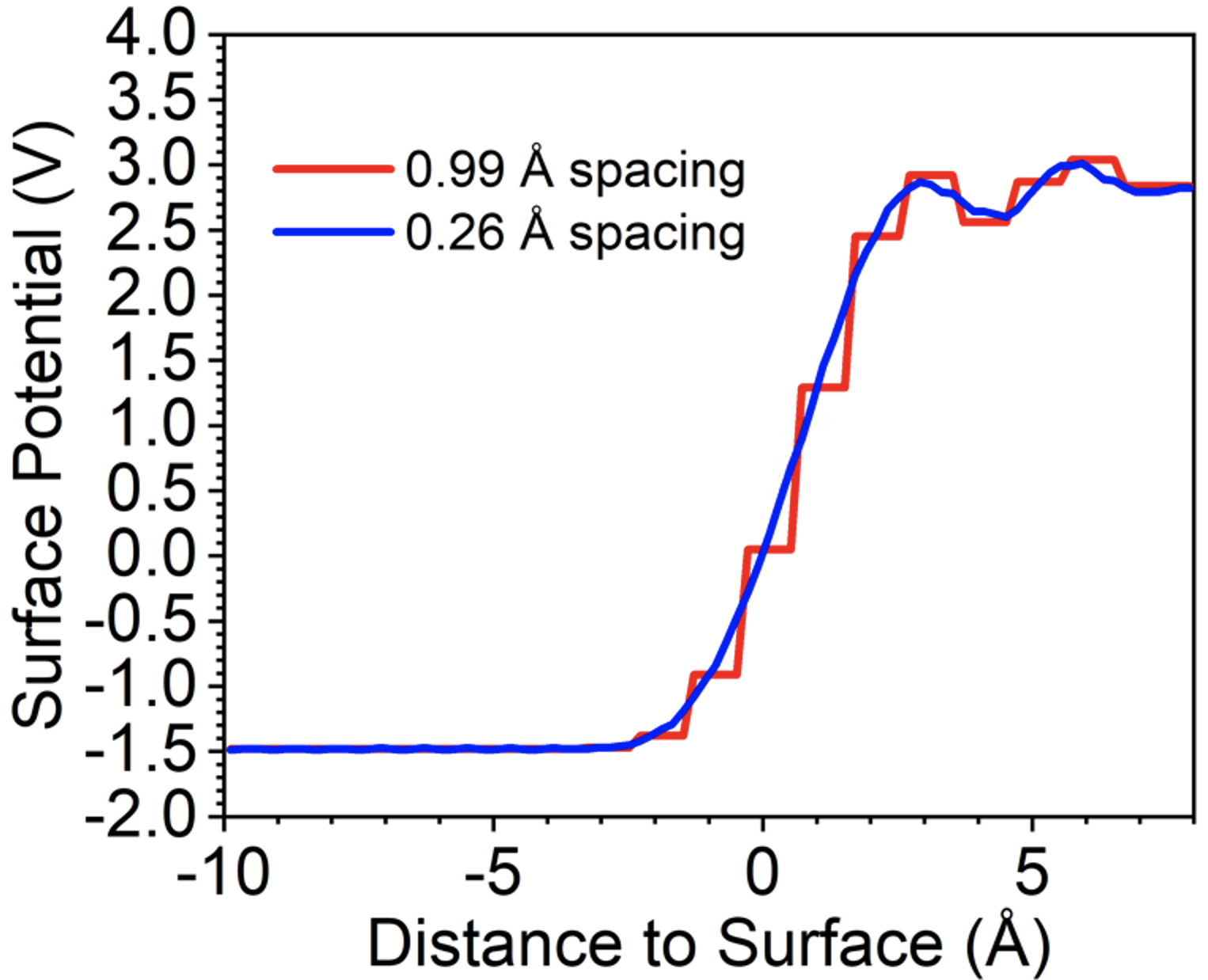

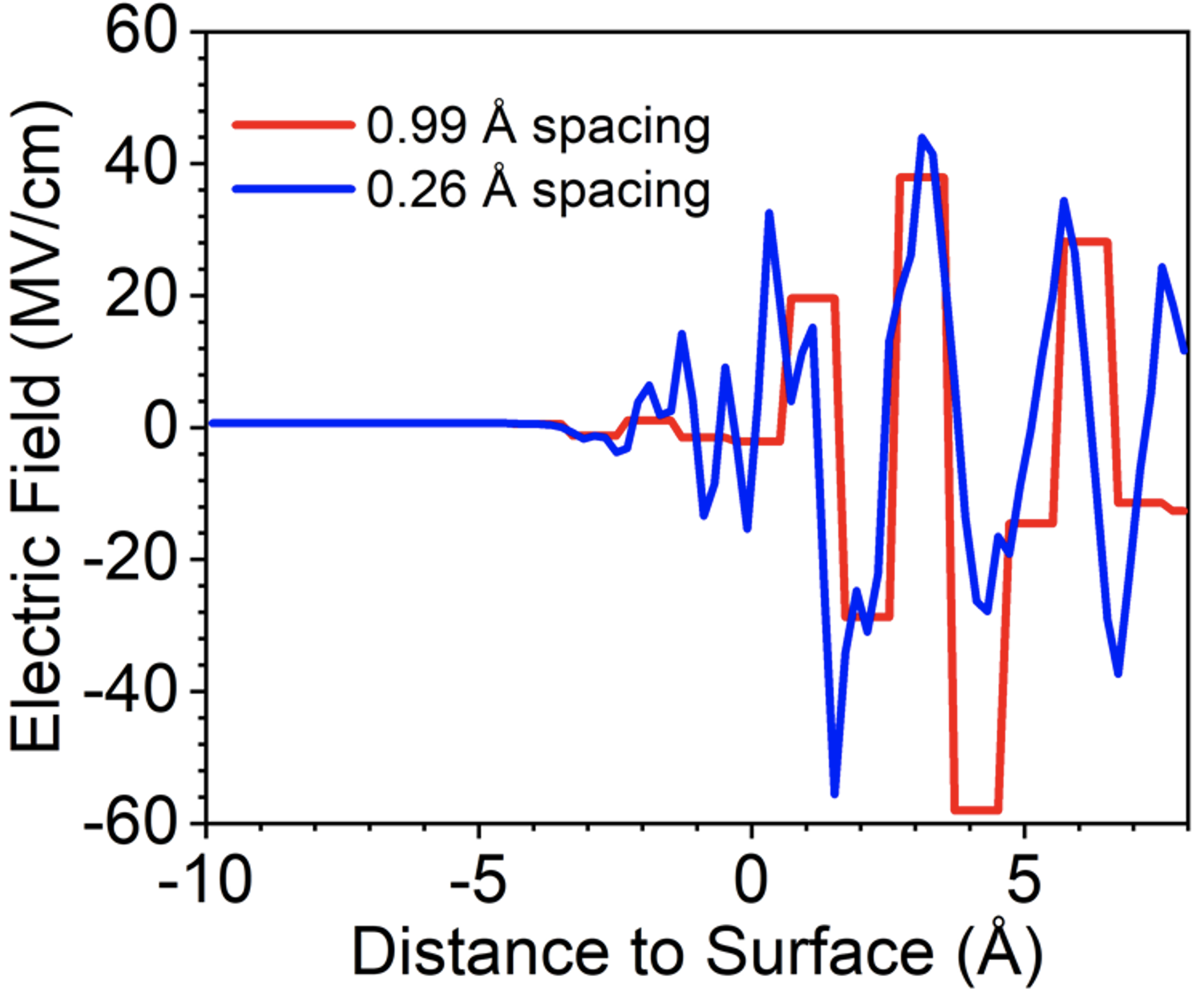


**Supplementary Figure 5.** *Evaluation of the surface potential and electric fields for a small slab geometry using different grid spacings for DFT/AIMD model.* (A) Surface potential at 0.99Å and 0.26Å spacing. Direct electric field calculations in the normal to surface direction for the small DFT system using (B) 0.99Å and (C) 0.26Å spacing. We used the B97M-rV functional^2, 3^ with the TZV2P basis set in CP2k^4^.


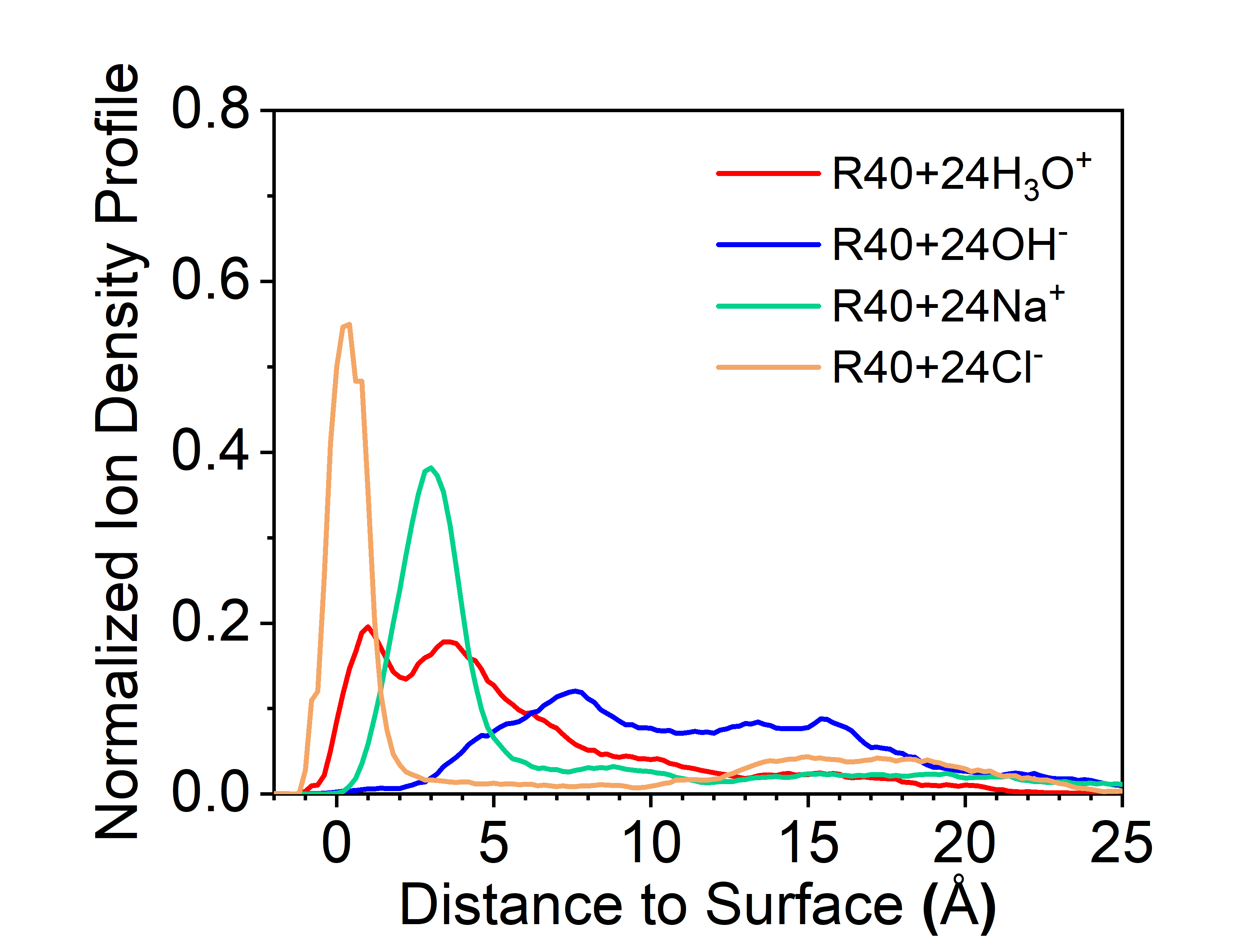


**Supplementary Figure 6.** *Ion density profiles.* The ion density profile of a R40 droplet with an excess of Na^+^ ions, Cl^–^ ions, H_3_O^+^ ions and OH^–^ ions.


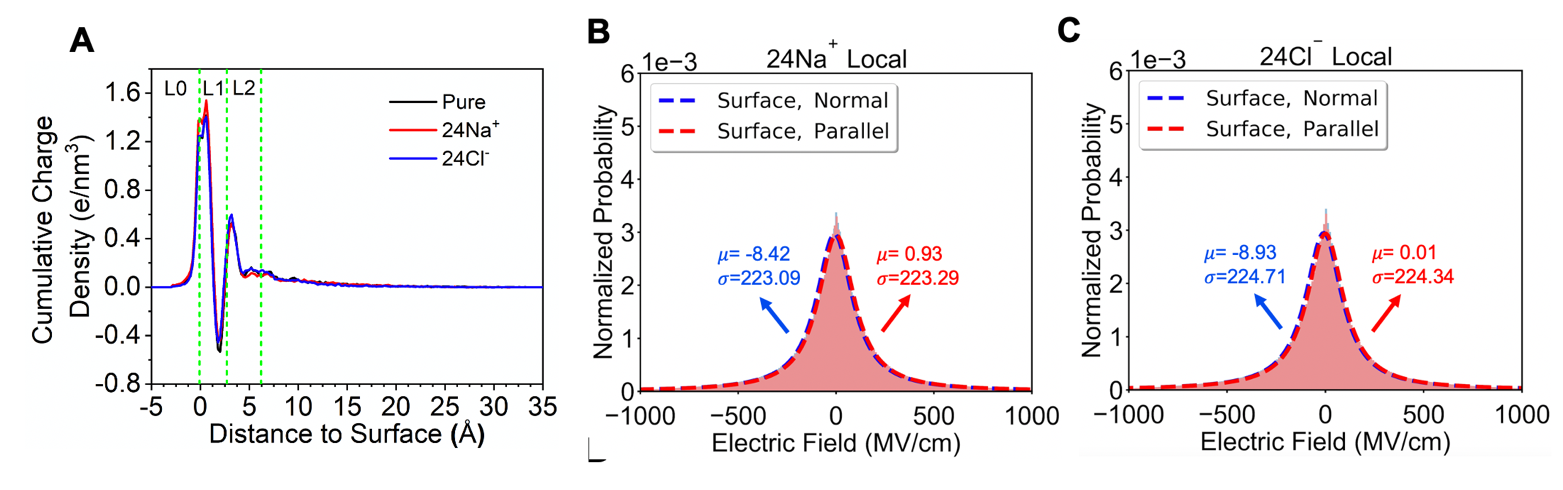


**Supplementary Figure 7**. *Interfacial electric fields of droplets in the presence of excess Na^+^ and Cl^–^ ions.* (A) Cumulative charge density for pure water and in presence of excess Na^+^ or Cl^–^. The Lorentzian signatures of the electric fields normal to the surface for the R40 water droplet with (B) Na^+^ and (C) Cl^–^ ions.


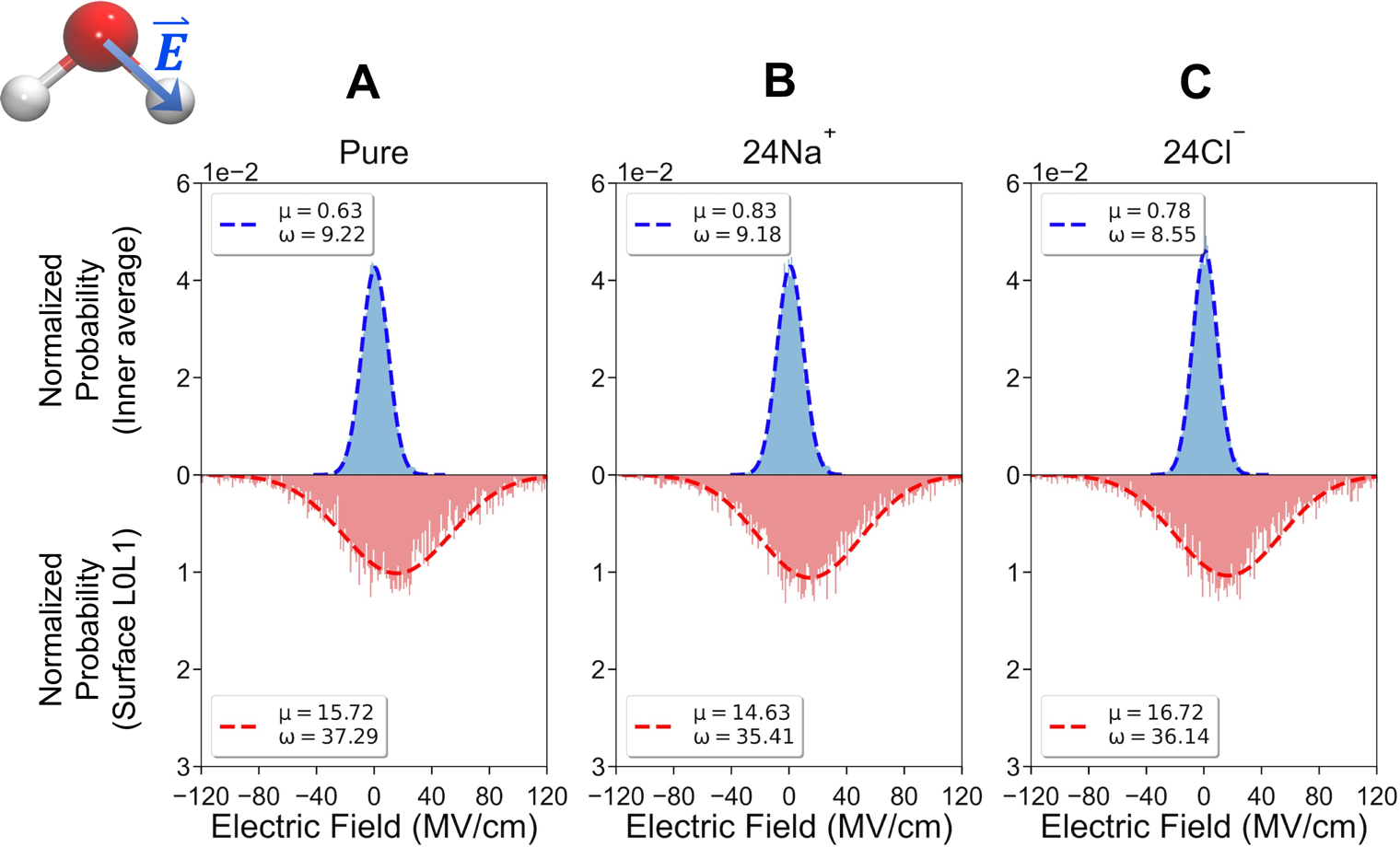


**Supplementary Figure 8.** *Electric field distributions arising from electric field projections on the O-H bonds of* *water.* The hydrogen-bonded water molecules in the inner droplet region (blue) and the surface free O-H water bonds (red) respectively for the R40 droplet with (A) pure water, (B) water with 24 Na^+^ ions, and (C) water with 24 Cl^–^ ions.

**Supplementary Table 1.** The analysis of the electric field and electrostatic potential for a single water molecule using DFT (0.99 Å grid spacing) and ReaxFF/CGeM (1 Å grid spacing). The averages are done over the grid points as a function of distance to atoms. We used the B97M-rV functional^2, 3^ with the TZV2P basis set in CP2k^4^.

**Supplementary Table 2.** The analysis of the electric field and electrostatic potential for a single water molecule using DFT (0.26 Å grid spacing) and ReaxFF/CGeM (0.25 Å grid spacing). The averages are done over the grid points as a function of distance to atoms. We used the B97M-rV functional^2, 3^ with the TZV2P basis set in CP2k^4^.

**Supplementary Table 3.** Table of averaged electric field with/out excess charges at surface and the bulk region defined by instantaneous surface method. Unit in MV/cm. Error is within 1MV/cm.

| **System** | **Instantaneous Surface Method** | |
| --- | --- | --- |
|  | **Surface** | **Inner bulk** |
| R40 pure | -8.87 | 1.15 |
| R60 pure | -8.61 | 0.91 |
| R80 pure | -8.76 | 0.89 |
| R40 + 24 H_3_O^+^ | -8.66 | 0.52 |
| R40 + 24 OH^–^ | -9.24 | 0.69 |
| R40 + 24 Na^+^ | -8.42 | 0.93 |
| R40 + 24 Cl^–^ | -8.93 | 0.01 |
| R40 + 16Na^+^ 8Cl^–^ | -8.57 | 0.84 |
| R40 + 8Na^+^16Cl^–^ | -8.68 | 0.64 |
| R40 + 12Na^+^12Cl^–^ | -8.67 | 0.80 |

**Supplementary References**

1. Cooper RJ, O'Brien JT, Chang TM, Williams ER. Structural and electrostatic effects at the surfaces of size- and charge-selected aqueous nanodrops. *Chem Sci* 2017, **8:** 5201-5213.

2. Mardirossian N, Head-Gordon M. Mapping the genome of meta-generalized gradient approximation density functionals: The search for B97M-V. *The Journal of Chemical Physics* 2015, **142**(7)**:** 074111.

3. Mardirossian N, Ruiz Pestana L, Womack JC, Skylaris C-K, Head-Gordon T, Head-Gordon M. Use of the rVV10 Nonlocal Correlation Functional in the B97M-V Density Functional: Defining B97M-rV and Related Functionals. *The Journal of Physical Chemistry Letters* 2017, **8**(1)**:** 35-40.

4. Hutter J, Iannuzzi M, Schiffmann F, VandeVondele J. cp2k: atomistic simulations of condensed matter systems. *WIREs Computational Molecular Science* 2014, **4**(1)**:** 15-25.
